# Video captioning based on vision transformer and reinforcement learning

**DOI:** 10.7717/peerj-cs.916

**Published:** 2022-03-16

**Authors:** Hong Zhao, Zhiwen Chen, Lan Guo, Zeyu Han

**Affiliations:** 1 School of Computer and Communication, Lanzhou University of Technology, Lanzhou, Gansu, China; 2 Network & Information Center, Lanzhou University of Technology, Lanzhou, Gansu, China

**Keywords:** Video captioning, Vision transformer, Reinforcement learning, Long short-term memory network, Computer vision, Natural language processing, Attention mechanism, Encode-decode, Deep learning

## Abstract

Global encoding of visual features in video captioning is important for improving the description accuracy. In this paper, we propose a video captioning method that combines Vision Transformer (ViT) and reinforcement learning. Firstly, Resnet-152 and ResNeXt-101 are used to extract features from videos. Secondly, the encoding block of the ViT network is applied to encode video features. Thirdly, the encoded features are fed into a Long Short-Term Memory (LSTM) network to generate a video content description. Finally, the accuracy of video content description is further improved by fine-tuning reinforcement learning. We conducted experiments on the benchmark dataset MSR-VTT used for video captioning. The results show that compared with the current mainstream methods, the model in this paper has improved by 2.9%, 1.4%, 0.9% and 4.8% under the four evaluation indicators of LEU-4, METEOR, ROUGE-L and CIDEr-D, respectively.

## Introduction

Generating video content description by manual annotation is time-consuming and inapplicable for large volumes of videos. With the continuous and rapid development of information technology, short video sharing platforms represented by Tiktok, Kwai and micro-blog have emerged. These platforms produce a large number of videos with various views and tendencies in the short term. There may be some videos that have a negative impact on users, and bring great hidden dangers if these videos contain blood, pornography, violence and other harmful information without timely review. Therefore, it is of great significance to adopt automatic means to timely review video content and reject undesirable videos in real time.

At present, video captioning methods are mainly based on template matching and deep learning (Guadarrama et al., 2013; Perez-Martin, Bustos & Pérez, 2021; Zhu, Duan & Yu, 2021). The template matching method first designs a fixed language template according to the video content structure. Then, the main object (noun), action (verb) and scene information are detected using target detection algorithms. Finally, the extracted video information is filled into the language captioning template to complete the captioning of one or more short videos. For example, Kojima, Tamura & Fukunaga (2002) establish the mapping relationship between visual objects or actions and specific concepts, and determine the corresponding syntactic components. It solves the cross-modal problem between video image and text description (Perez-Martin, Bustos & Pérez, 2021). With the help of the and-or diagram template, Gupta et al. (2009) generate a single captioning for each action according to the movement sequence relationship of the people in the video, and combines all the single captions based on the simulated video content to form a paragraph captioning for the video. These methods focus on action semantics and break through the limitation of only outputting action descriptions based on video content in the original video action recognition task. However, their captioning of other components lack flexibility. Therefore, in order to be more consistent with the conventions of natural language, Rohrbach et al. (2013) imitate machine translation method and construct an encoding-decoding framework to generate a more flexible captioning. Likewise, Xu et al. (2015) use Word2Vec to extract the features of the captioning sentence while using a neural network to encode video features, and jointly embed the visual features and the captioning features to improve the accuracy of the captioning.

Video captioning methods based on deep learning are inspired by the encoder-decoder framework used in machine translation research, and many similar video captioning models have been designed. For example, Yadav & Naik (2021) use Deep-LSTM and Bahdanau attention mechanism as the encoder and decoder of the model to generate captions. Alkalouti & Masre (2021) exploit an encoder-decoder structure that combines two deep learning algorithms, YOLO and LSTM, to automatically generate video captioning. Later on, on the basis of deep learning, the literatures (Aafaq et al., 2019; Chen et al., 2019a; Zhang & Peng, 2020; Zhang et al., 2019) extend the research on video content description by using RNN sequence features and 3D convolution features.

The most existing video captioning methods utilize deep convolutional neural networks or 3D convolutional neural networks as encoders to extract visual representation vectors. It decodes its visual representation vector as the input of Recurrent Neural Network (RNN) to generate serialized natural language expression. For example, Venugopalan et al. (2015) design a set of S2VT models, extract video features and optical flow features through the DCNN model, and make use of two LSMT networks for feature encoding and decoding to generate the final captioning. Based on the S2VT framework, Tang, Wang & Li (2019) combine the residual mechanism, multi-structure LSTM sequence fusion and visual feature complementation to further optimize the model. It effectively solves the redundancy of the CNN features of optical flow frames for modeling video static features and dynamic sequence features, and improves the expression ability of the model. In addition, Bin et al. (2016) design a bidirectional LSTM network to extract the time-series features of the video from the front and rear dimensions and fuse the features and frame-level sequence features during the training process, which enhances the expressive ability of the model. However, it is difficult for a single video description task to fully extract the timing information and logical dynamic information in the video. To tackle this problem, Pasunuru & Bansal (2017a) propose a multi-task learning method, which uses video prediction task to learn more video context knowledge and semantic information. Although this method improves the coherence of description sentences, it has poor generalization performance for complex multi-scene tasks. Inspired by reinforcement learning, Pasunuru et al. use the reward mechanism of reinforcement learning as a benchmark model to improve the accuracy of video content description and the coherence of language description. Since then, the literature (He et al., 2019) focuses on improving the sentence components of the description sentences, and guide the model to generate words according to the Part-of-Speech (POS). Zheng, Wang & Tao (2020) emphasize the relevance of subject, predicate and visual scene in sentences, and built a SAAT model based on Transformer and verify the validity of the model.

Although the use of feature sequences extracted by RNN to establish a language description model is successful, 3D convolutional networks can extract both spatial and temporal features of videos, which can further strengthen the features of static visual semantic objects and dynamic visual events in videos and improve the robustness of the model. For example, Yao et al. (2015) apply a 3D convolutional network to extract video features and introduce an attention mechanism. It assigns weights of 3D spatiotemporal feature assignments at different time steps to guide descriptive sentence generation (Yao et al., 2015). Inspired by the human visual tracking mechanism, Yu et al. (2017) propose an attention network based on gaze tracking coding. The network improves the description accuracy of the model by integrating the visual tracking mechanism in the attention model (Yu et al., 2017). However, the model uses GRU to distribute attention weight in time domain, which easily leads to long-term dependence and semantic misplacement of multi-modal information. In view of this, Wang et al. (2018) propose a multi-modal memory model that closely combines visual and linguistic information to improve the accuracy of words used in generated sentences. Pei et al. (2019) utilize 2D and 3D convolution features of videos and adopt attention mechanism for feature fusion, and predict words on each time step by constructing GRU network. Similarly, Chen et al. (2019b) integrate multi-model features including 2D and 3D convolution features and MFCC (Mel frequency Cepstrum coefficient) audio features to expand the feature dimension and fully mined video information.

In summary, although the video captioning method based on template matching is simple and straightforward, these methods depend too much on the preset templates and rules. It leads to monotonous captioning sentences and poor flexibility. Although the video captioning method based on deep learning can effectively solve the above problems, the common video captioning method usually directly takes the final output state of encoding as the input in the decoding stage, and loses a large number of intermediate hidden states.

In recent years, inspired by the successful application of Transformer in vision tasks in natural language processing, a large number of vision tasks also use Visual Transformer (ViT) as a model encoder to verify the efficiency of the model, such as semantic segmentation, image editing and entity segmentation. Therefore, the paper proposes a video content description method integrating vision transformer and reinforcement learning. It utilizes the transformer encoder provided by ViT as the feature encoder, and globally encodes the video features combined with the hidden state in the middle of the encoder. It solves the problem that the traditional encoder loses the information of the middle-hidden layer and cannot globally encode the video features. The main work of this paper is following:

(1) Use an encoder composed of Transformer Encoder blocks to encode video features in a global view, thereby reducing the loss of intermediate hidden layer information.
(2) Introduce the Policy Gradient reinforcement learning method to improve the accuracy of the model.
(3) Conduct Experiments on the MSR-VTT dataset to demonstrate the effectiveness of the video captioning method proposed in this paper.

## Video Content Captioning Model

### Model structure

**Figure 1:**
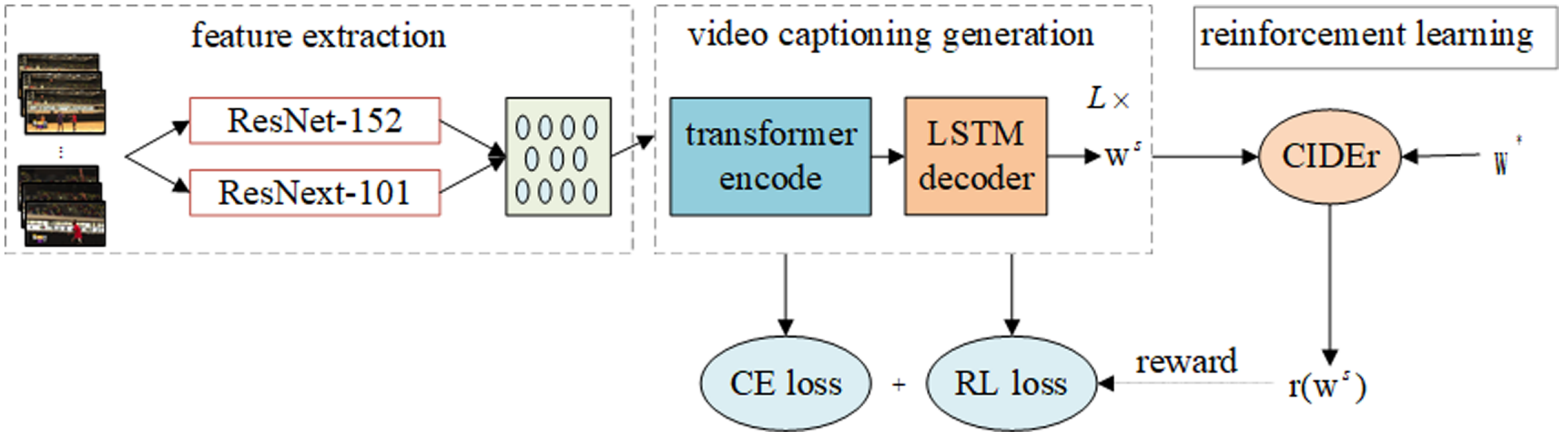
Video content captioning model structure. The model includes three parts: feature extraction, video captioning generation, reward mechanism (Policy Gradient). *L* is the number of transformer encoder blocks in the encoder of the model, *w^s^* is the word sequence generated by the model, and r(*) is the reinforcement learning reward function.

As shown in Fig. 1, the model includes three modules: feature extraction, video caption generation and reinforcement learning feedback mechanism. The feature extraction module extracts the features of the segmented video frame through a convolution neural network. The video caption generation module adopts an encoding-decoding framework to encode the features before decoding to generate the caption of the video content. The reinforcement learning feedback mechanism module takes the model as the agent, the video data and the real caption as the environment, and optimizes the caption of the video content based on the CIDEr index.

### Feature extraction

Video data is composed of objects, scenes, people and other static elements in the spatial domain, and its structure is composed of multiple continuous video frames. There are changes in motion trajectories between frames, which contain rich temporal motion information (Wang, 2020). Hence, both frame level static features and temporal motion features of videos need to be extracted.

In deep learning methods, we usually extract richer features by continuously stacking the number of network layers. However, the accuracy of the model gradually begins to saturate and rapidly decline as neural networks deepen. It will lead to the disappearance of the gradient and the degradation of accuracy. In response to this problem, He Kaiming et al. designed ResNet network and introduced deep residual learning module (Ioffe & Szegedy, 2015). It fits the residual mapping by stacking layers, so that the accuracy of the model increases as the number of network layers increases. In addition, the deeper network layers will increase the computational complexity of the model. However, ResNeXt network can effectively solve such defects. To sum up, ResNet and ResNeXt (Xie et al., 2017) networks are selected to extract static features and temporal motion features in the video. To be clear, we used the ResNeXt pretraining model trained on Dataset Kinetics (Carreira & Zisserman, 2017).

Before extracting the video features, we first segment the video data into $224 \times 224$ video frames using FFMPEG tool. We do not limit the number of video frames, but uniformly process 50 frames of equal length before inputting the reference model. Then, all video frames are fed into the feature extraction network to obtain the complete features of the video. Suppose that the video is segmented into N video frames, and the frame sequence is ${\rm V} = \left\{ {{X_i},y} \right\}_{i = 1}^N$. We extract 2,048-dimensional static feature *r*_*i*_ and dynamic feature *e*_*i*_ for each frame respectively. The sum result *x*_*i*_ of the two features is used as the overall feature of the video. The sequence of visual features is ${x_v} = \left\{ {{x_1},{x_2},{x_3}, \cdots ,{x_N}} \right\}$, which is calculated as Eqs. (1)–(3).

(1) $${r_i} = {f_r}({X_i})$$

(2) $${e_i} = {f_e}({X_i})$$

(3) $${x_i} = {r_i} + {e_i}$$

where *r*_*i*_ represents the result of static feature extraction, *e*_*i*_ is the result of temporal motion feature extraction, *f*_*r*_ and *f*_*e*_ represent static and dynamic feature extraction functions respectively, ${X_i} \in {R^{C \times H \times W}}$ represents the *i*-th video frame, ${x_i} \in {R^{{d_{visiual}}}}$, *d*_*visiual*_ is the dimension of the video feature. We set the size of *d*_*visiual*_ to 4,096 dimensions. *C*, *H* and *W* are the number of channels, height, and width of the video frame. Their values are 3, 224, and 224.

### Video captioning generation

**Figure 2:**
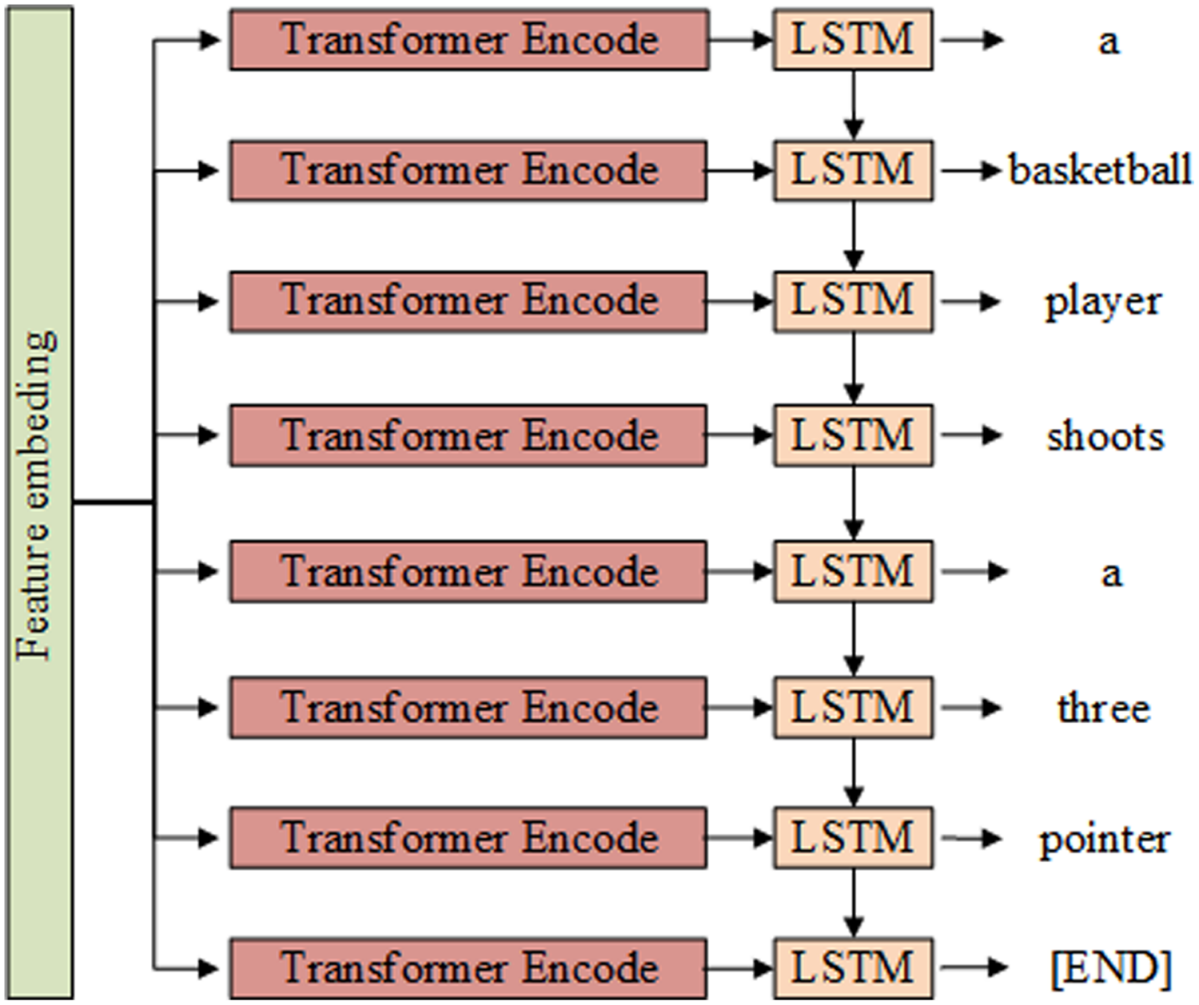
Captioning content generation module structure composed of Transformer and LSTM.

The video caption generation phase consists of the encoding and decoding of the video features, as shown in Fig. 2. In the encoding stage, the embedded feature vectors are fed into the encoder to encode the video features globally. In the decoding stage, the encoding result is taken as the input to the decoder, and its output is the video captioning statement.

#### Encoder

The vision transformer (ViT) model proposed in 2021 can encode image features with the global field of view, and tackle the problem that convolution networks are highly sensitive to the high-frequency information in the image. Inspired by literature (Mubashira & James, 2020), we use the transformer encoder of ViT as the model encoder, and effectively use the intermediate state of the encoder to implement the global encoding of video features.

**Figure 3:**
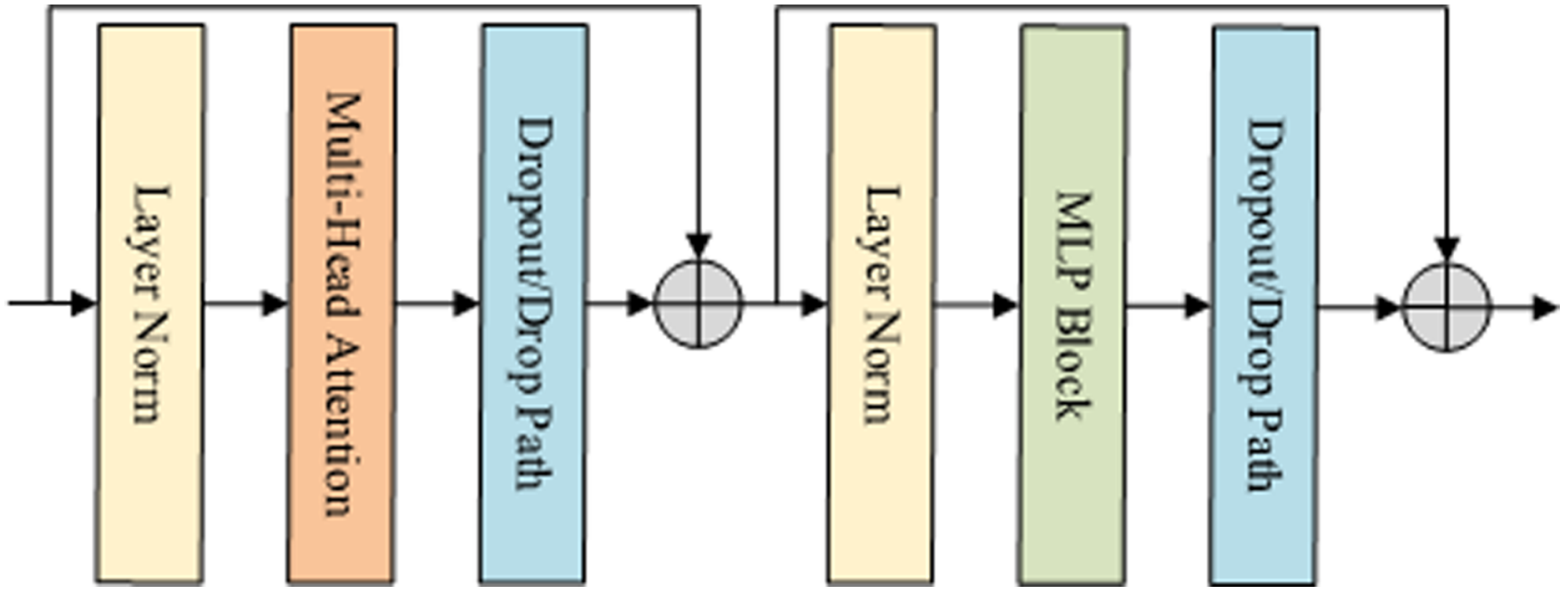
Encoder block structure in VIT. Specifically, the encoding block first inputs the features into Layer Norm and Multi-Head Attention, and then sends them to the MLP block.

The encoder of the benchmark model is made up of a stack of 12 single Vision Transformer encoding blocks. Each block consists of Multi-Head Attention (MHA) and MultiLayer Perceptron (MLP) Block, as shown in Fig. 3. To ensure the stability of the distribution of data features, the data is normalized by Layer Norm (LN) before each block is executed.

In the *x*-th time step shown in Fig. 3, it is assumed that the video feature extracted by the convolution network is *C*. First, we use MHA function to calculate the normalized characteristic *C* of the previous time step. Then, apply the MLP function to calculate the output of the coding block. Finally, the *N* features are normalized. The result is expressed as the final feature of the encoder, and its output size is 1,024 dimensions, which is calculated as Eqs. (4)–(6).

(4) $$\matrix{ {z_l^{\prime} = MSA(LN({z_{l - 1}})) + {z_{l - 1}}}  {l = 1 \ldots N} \cr }$$

(5) $$\matrix{ {{z_l} = M{\rm LP}(LN(z_l^{\prime})) + z_l^{\prime}} & {l = 1 \ldots N} \cr }$$

(6) $$\matrix{ {{x_l} = LN({z_l})} & {l = 1 \ldots N} \cr }$$

where $z_l^{\prime}$ represents the output of the multi-head attention mechanism, *z*_*l*_ is the output of the multilayer perceptron, *x*_*l*_ is the output of the encoder at time *l*, which is the result of the global feature encoding. *N* is the total time step length.

#### Decoder

**Figure 4:**
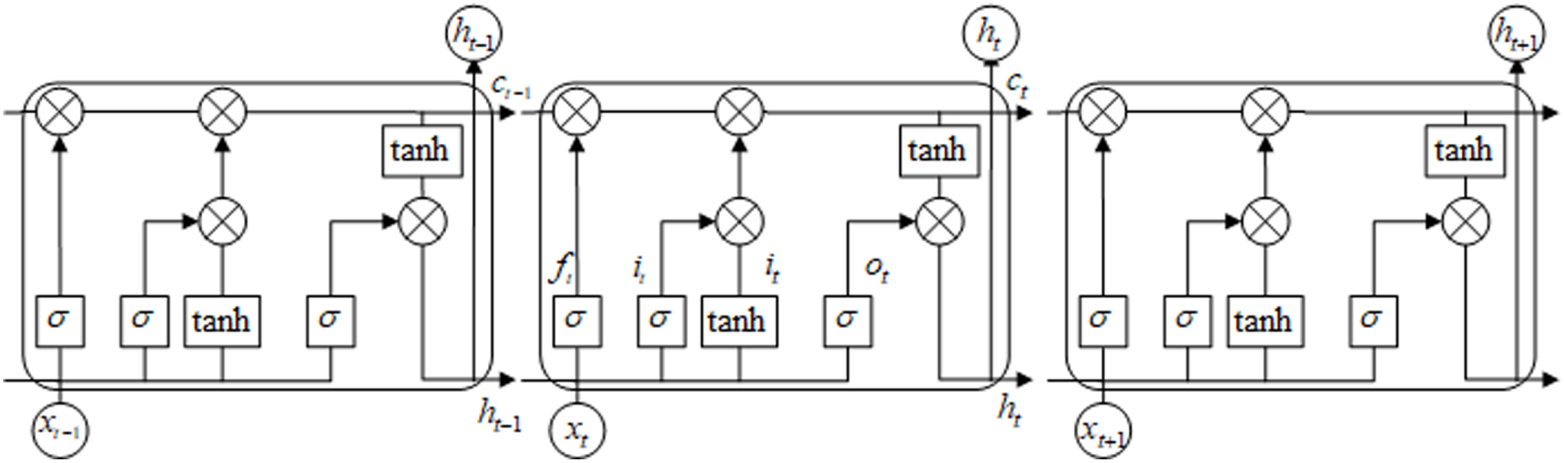
LSTM network model.

Taking into account the timing relationship between video frames, the benchmark model in this paper uses a multi-layer Long Short-Term Memory neural network (LSTM) to construct the decoder. The LSTM consists of input gate, forgetting gate, output gate and memory unit. The network structure of the LSTM unit is shown in Fig. 4. The LSTM network transmits cell state as well as hidden state in forward propagation. This effectively solves the problem that the parameters of other recurrent neural networks cannot be continuously optimized due to the disappearance of gradient in the process of back propagation (Yang et al., 2018). Therefore, we construct an LSTM decoder to remember the context timing relationship while retaining the video content information, and generate a more logical caption.

As shown in Fig. 4, let the coding result of the *t* time model be 1,024-dimensional vector *x*_*t*_, the hidden layer feature corresponding to the input feature be *h*_*t*−1_, and the cell memory unit of the LSTM network be *c*_*t*_. Then the activation function $\sigma$ is used to obtain the input feature vector *i*_*t*_ of the LSTM unit. Similarly, the forgetting feature *f*_*t*_ and the output feature *o*_*t*_ can be obtained as Eqs. (7)–(12).

(7) $${i_t} = \sigma ({W_{xi}}{x_t} + {W_{{\rm h}i}}{h_{t - 1}} + {b_i})$$

(8) $${f_t} = \sigma ({W_{xf}}{x_t} + {W_{{\rm h}f}}{h_{t - 1}} + {b_f})$$

(9) $${o_t} = \sigma ({W_{xo}}{x_t} + {W_{{\rm ho}}}{h_{t - 1}} + {b_o})$$

(10) $${g_t} = \phi ({W_{xg}}{x_t} + {W_{{\rm hg}}}{h_{t - 1}} + {b_g})$$

(11) $${c_t} = {f_t} \odot {c_{t - 1}} + {i_t} \odot {g_t}$$

(12) $${{\rm h}_t} = {o_t} \odot \phi ({c_t})$$

where $\odot$ is the Hadamard product operation, *i*_*t*_ represents the inputs, *f*_*t*_ is the forgetting feature, *o*_*t*_ is the output gate, *g*_*t*_ is the input modulation gate, *W* and *b* are the parameters to be optimized. We use the sigmoid activation function and the tanh activation shown in Eqs. (13) and (14). Adding forget gates and memory gates in the decoding process will enable the video captioning model to memorize the video content in the time domain, and generate a more logical caption.

(13) $$\sigma (x) = \displaystyle{1 \over {1 + {e^{ - x}}}}$$

(14) $$\phi (x) = \displaystyle{{{e^x} - {e^{ - x}}} \over {{e^x} + {e^{ - x}}}}$$

The detailed process of the video feature decoding is shown in Table 1.

**Table 1:** The detailed process of the video feature decoding.

Algorithm 1: Video feature decoding	
**Inputs**	Initialize weights ${W_{hi}}$, ${W_{xi}}$ and encoded feature ${x_t}$
1	Calculate the forgetting gate eigenvector ${f_t}$ using Eq. (8);
2	Use Eq. (7) to calculate the input gate feature vector ${i_t}$;
3	Calculate the input modulation gate feature vector *g_t_* using Eqs. (10) and (11), and update *c_t_*_−1_ to *c_t_*
4	Calculate the output gate eigenvector *O_t_* using Eq. (9);
5	Repeat steps (1) to (4) until all features are decoded, and the input of the last decoding unit is the output result of the decoder
**Output**	Decoded feature vector

Furthermore, in order to enable mapping between the decoded result and the text, we preprocess the captioning text tags corresponding to the video. First, we use the word embedding method to encode each word in the caption into a 512-dimensional vector ${y_t} \in Y({y_1},{y_2},{y_3}, \ldots ,{y_{512}})$. We use a dataset in which the maximum length of all captions is 20 words. Therefore, we set the length of the video captioning sentence to 20. When embedded, captions of less than 20 words are represented by the number 0. Assuming the caption generated by the model be expressed as $y_t^{\prime} \in {Y^{\prime}}(y_1^{\prime},y_2^{\prime},y_3^{\prime}, \ldots ,y_{\rm m}^{\prime})$, then the conditional probability representation of ${Y^{\prime}}$ with respect to *X* is shown in Eq. (15).

(15) $$P({Y^{\prime}}\left| {X,Y} \right.) = P(y_1^{\prime}, \cdots ,y_m^{\prime}\left| {{x_1}, \cdots ,{x_n};{y_1}, \cdots ,{y_n}} \right.) = \prod\limits_{t = 1}^m {P(y_t^{\prime}\left| {{h_{n + t - 1}},y_{t - 1}^{\prime}} \right.)}$$

where the conditional probability $P(y_t^{\prime}\left| {{h_{n + t}})} \right.$ represents the probability value of all words in the *corpus* corresponding to the softmax layer. We represent the string [END] as the end of the sentence. The end marker not only prompts the model to switch coding and decoding in the training stage, but also can be used as a marker to describe the completion of generation in the test stage.

In summary, at time step *t*, the model first encodes the video features through the transformer encoder block, and then sends it to the multi-layer LSTM network to decode and generate the caption $y_t^{\prime}$. Each time a caption is generated during the training process, the model calculates the cross-entropy loss based on the generated sentence and the real captioning, and continuously updates and optimizes the model parameters. The calculation is shown in Eqs. (7)–(12).

### Reinforcement learning optimization

**Figure 5:**
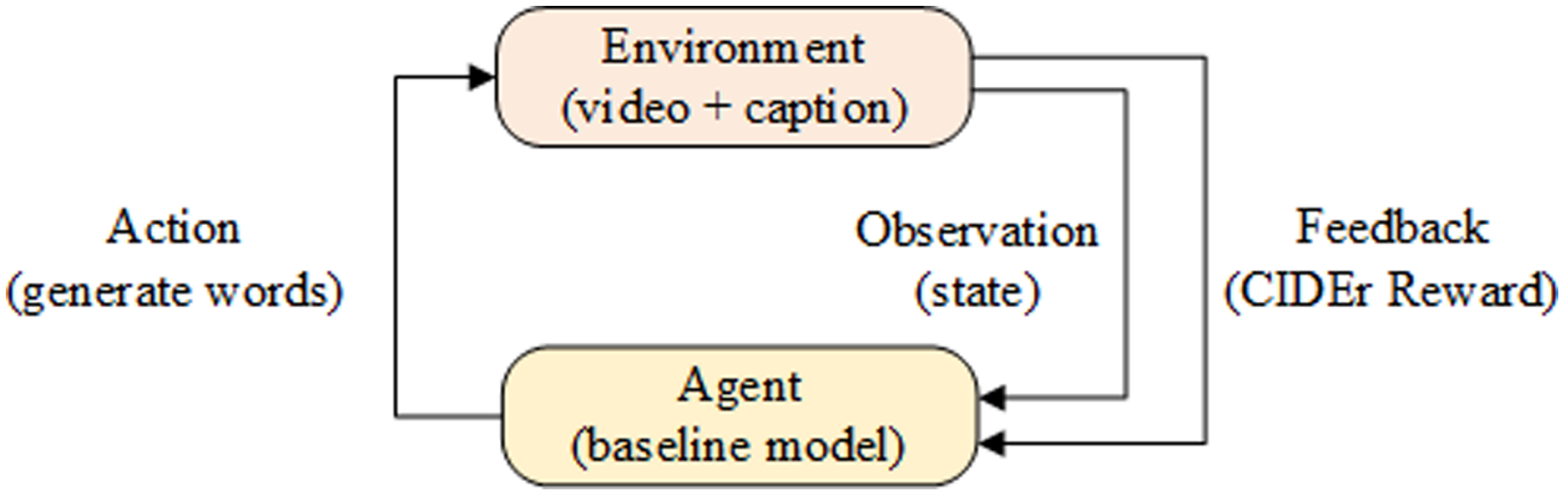
Reinforcement Learning network model.

In order to improve the accuracy of video captioning model, a reinforcement learning method is introduced to learn the strategy gradient ${\pi _\theta }$, where $\theta$ represents the model parameters. Specially, benchmark models were used as Agent, video and captioning as Environment in reinforcement learning. In each time interval of the model, Agent generates a word accordingly. When the generated word is the end-of-sequence token [END], Environment calculates the reward value R(t) nd feeds it back to the Agent. The model optimization process is shown in Fig. 5.

When optimizing the model with reinforcement learning, let the sequence of words, status values and rewards generated by a video be $\tau = \left\{ {{s_1},{a_1},{r_1},{s_2},{a_2},{r_2}, \cdots ,{s_t},{a_t},{r_t}} \right\}$, where *s*_*t*_ represents the status of the Environment at time *t*, at is the word generated at time *t*. Finally, the model calculates the loss function gradient and optimizes the model parameters according to the loss value and the reward value as follows:

(16) $$L(\theta ) = - \displaystyle{1 \over N}\sum\limits_\tau {R(\tau )} \log {\pi _\theta }(\tau ) = - {E_{\tau\ \sim\ {\pi _\theta }}}[R(\tau )]$$

(17) $${\nabla _\theta }L(\theta ) = - {E_{\tau\ \sim\ {\pi _\theta }}}[R(\tau ) \cdot \nabla \log {\pi _\theta }(\tau )]$$

where ${\pi _\theta }(\tau )$ is the probability that the model is generated and described as $\tau$, and *N* is the number of samples. In order to improve the readability and fluency of the generated captions, we refer to the literature (Pasunuru & Bansal, 2017b) using the method of mixed loss in reinforcement learning to enhance learning. The proportion of cross entropy loss *L*_*ce*_ and reinforcement learning loss *L*_*rl*_ is adjusted by super parameter $\gamma$. Reinforcement learning is expressed as shown in Eq. (18).

(18) $${L_{mix}} = (1 - \gamma ){L_{ce}} + \gamma {L_{rl}}$$

In video captioning, traditional reward methods include CIDEr, BLUE and METEROR. Among them, CIDEr is a weighted evaluation index, which pays more attention to whether the generated captions contain the focus of the image content. The evaluation index is more consistent with the human evaluation method. Consequently, that we used cider as the reward index of reinforcement learning.

## Experimental Results and Analysis

### Datasets and evaluation indicators

We chose the MSR-VTT dataset commonly used in the field of video captioning, which contains 10,000 videos. Each video in the dataset contains 20 manually annotated reference captions, which are partitioned by Xu et al. (2016) before training. Specifically, it is divided into 6,513 as training data and 497 as verification data, and the rest as test data. In addition, we extract video features through the concept-v4 network proposed by Szegedy et al. (2017). The English annotation sentences in the above dataset were selected for model training.

Four common evaluation indicators of ROUGE-L, METEOR, BLEU-4 and CIDEr-D were used when evaluating the model (Denkowski & Lavie, 2014; Lin, 2004; Papineni et al., 2002; Vedantam, Lawrence Zitnick & Parikh, 2015). The ROUGE-L index considers the order of words in sentences and evaluates the meaning of sentences. The METEOR indicator is based on the single-precision weighted harmonic average and the single word recall rate. The evaluation results of this indicator are more relevant to the results of manual evaluation. Bleu-4 index measures the semantic similarity between the generated result and the target sentence by defining the number of 4-gram. The CIDEr index is often set as an evaluation method in the field of image or video captioning. The method represents a caption generated by the model and a real caption as a word frequency vector and inverse word frequency vector, and uses cosine similarity to measure the captioning performance. The evaluation index has higher reference value in the field of video and image content captioning (He et al., 2020). The higher the percentage score of these four standard evaluation indicators, the closer the generated captioning semantics to the real captioning.

### Parameter setting

During feature extraction, videos will be randomly segmented into $224 \times 224$ frames. A feature vector with 4,096 dimensions corresponding to the number of frames will be obtained. Then process all the feature data into the same dimension $50 \times 4,\!096$. The LSTM decoder of the baseline model has a hidden layer size of 1,024. Before inputting the video features into the model, the 4,096-dimensional feature is mapped to 1,024-dimensional, and the word embedding is expressed as a 512-dimensional vector. The experiment uses the Adam optimizer to train the network, and the initial value of the learning rate is set to 0.0001. It can be reduced with the iteration of training. To prevent overfitting, we introduce the vertical connection dropout method proposed by Zaremba, Sutskever & Vinyals (2014), which can achieve the regularization effect. The initial value of all weights that need gradient update is set to a uniform distribution on the interval [−0.08, 0.08]. The width size of Beam Search is 5 in the testing phase.

### Result Analysis

In order to verify the efficiency of the video content description model in this paper, the current mainstream video description models are constructed under the same dataset and evaluation index system. Among them, the POS-CG uses both a POS sequence generator and a description generator. It uses reinforcement learning to optimize the model end-to-end (Wang et al., 2019). SAAT model strengthens the focus on predicates and actions in sentences and enhances the recognition of action words. It gives more consideration to the interaction between objects within the video (Zheng, Wang & Tao, 2020). Therefore, improve the logic and readability of the description. The Cident-RL model uses a mixture of cross entropy loss and reinforcement learning loss. It adds Entailment score into the reward mechanism to improve the readability of generated description (Pasunuru & Bansal, 2017b). The SGN model mines the semantic information in consecutive video frames and divides the video segments into units of different information according to the semantics (Ryu et al., 2021). The experimental results are shown in Table 2.

**Table 2:** Score comparison between models. Transformer-LSTM and Transformer-LSTM-RL denotes the results implemented by ourselves.

Model	B	M	R	C	loss
POS-CG (Wang et al., 2019)	38.3	26.8	60.1	43.4	XE
POS-CG (Wang et al., 2019)	39.6	27.5	61.3	50.8	RL
SAAT (Zheng, Wang & Tao, 2020)	40.5	28.2	60.9	49.1	XE
SAAT (Zheng, Wang & Tao, 2020)	39.9	27.7	61.2	51.0	RL
Cross-Entropy (Pasunuru & Bansal, 2017b)	38.6	27.7	59.5	44.6	XE
CIDEr-RL (Pasunuru & Bansal, 2017b)	39.1	28.2	60.9	51.0	RL
CIDEnt-RL (Pasunuru & Bansal, 2017b)	40.5	28.4	61.4	51.7	RL
SGN(G) (Ryu et al., 2021)	37.3	26.8	58.2	41.2	XE
SGN(V) (Ryu et al., 2021)	37.8	27.0	58.3	41.9	XE
SGN(R152) (Ryu et al., 2021)	39.6	27.6	59.6	45.2	XE
SGN(R101+RN) (Ryu et al., 2021)	40.8	28.3	60.8	49.5	XE
Transformer-LSTM	38.6	27.9	60.2	44.6	XE
Transformer-LSTM-RL	**42.0**	**28.8**	**62.0**	**54.2**	**RL**

**Note:**

The best results are in bold.

It can be seen from Table 2 that the method in this paper obtains BLEU-4 of 42.0, METEOR of 28.8, ROUGE-L of 62.0 and CIDEr-D of 54.2, respectively. Compared with the best SGN model among all comparison models, the BLEU-4 indicator is improved by 2.9%; Compared to the top-scoring model CIDEnt-RL on metrics such as METEOR, ROUGE-L and CIDEr-D, our method scores 1.4%, 0.9% and 4.8% higher, respectively. The reason is that the model in this paper inputs the extracted features into the encoder composed of Vision Transformer after extracting features from the video using CNN network. It can pay attention to local information and consider global features. In addition, the introduction of LSTM networks preserves contextual timing information, which makes the generated description more logical.

**Figure 6:**
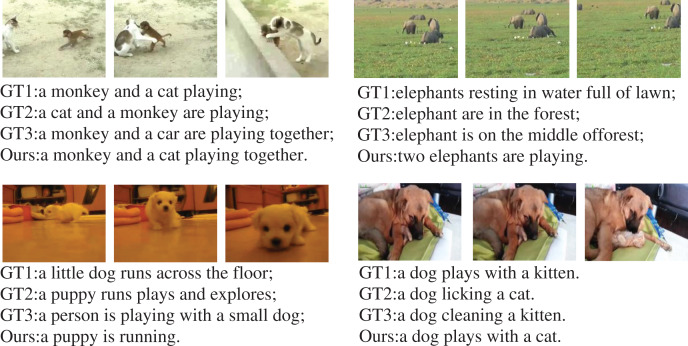
Visual comparison of video content captioning examples generated by our model. GT means real annotated captioning. Puppy image source: https://www.youtube.com/watch?v=AOjL8AoGzIg; Monkey and cat image source: https://www.youtube.com/watch?v=YvF-ZTH28yI; Elephants image source: https://www.youtube.com/watch?v=t1vzDlYTt1M; Dog and cat image source: https://www.youtube.com/watch?v=8-24M3tdxe8.

Video content captioning aims to generate more consistent captioning of video content. Figure 6 shows some examples of our model in the MSR-VTT dataset. Each video data in the figure lists three manually annotated captions and one model-generated captioning. The model in this paper has the ability to generate more accurate and more readable captions compared the captions marked manually. The reason is that the model pays more attention to the global feature of the video, so that it can consider the overall structure of the video. In addition, manual annotation captions are often limited to personal knowledge domains, interests, and language skills. As a result, the model can generate better captioning, which also verifies the effectiveness of the proposed method.

### Ablation experiment

In order to verify the advantages of the encoder module and reinforcement learning in the video content captioning model, we completed an ablation experiment on the MSR-VTT dataset. In detail, the LSTM-LSTM model generates video content captioning by using the LSTM network as encoder and decoder. LSTM-LSTM-RL integrates reinforcement learning on this basis, so that the weight parameters of the model are further optimized. Compared with the original model LSTM-LSTM, the model has increased by 0.5%, 0.5%, 1.4% and 6.4% respectively under the four evaluation indexes. The experimental results are shown in Table 3. It proves the effectiveness of reinforcement learning to optimize video content captioning model. However, both of them have the disadvantage of directly taking the final hidden layer state of the encoder as the input of the decoder. The models lose the content of the middle-hidden layer of the encoder, resulting in low model scores. Transformer LSTM model solves the problem, which replaces the encoder with vision transformer coding block. The model can globally encode video features and make full use of coding results in the decoding stage. Compared with the LSTM network as the encoder, the model with the Vision Transformer coding block as the encoder has achieved significant better results. The experimental results correspond to the Transformer-LSTM-RL are shown in Table 3. In summary, updating the encoder and introducing a reinforcement learning method improves the accuracy of the video content captioning task.

**Table 3:** Scoring results of ablation experiments.

Model	BLEU-4	METEOR	ROUGE-L	CIDEr-D
LSTM-LSTM	38.6	27.7	59.5	44.6
LSTM-LSTM-RL	39.1	28.2	60.9	51.0
Transformer-LSTM	38.6	27.9	60.2	44.6
Transformer-LSTM-RL	42.0	28.8	62.0	54.2

## Conclusions and Future Work

We propose a new video content captioning method based on VIT and reinforcement learning. We use the Transformer Encoder block of the VIT in the encoder, focusing on the overall structure of the video content. In addition, we use reinforcement learning and reward value from environment (captioning text and video) to optimize model parameters and improve the captioning performance. Multiple experiments on the MSR-VTT data set demonstrate the effectiveness of the proposed method measured by the evaluation indicators of METEOR, BLEU, ROUGE-L and CIDEr.

In the video captioning task, the collection and labeling of training data often consumes a lot of manpower and material resources. Therefore, in the future work, the zero-shot and few-shot learning techniques can be used to achieve adequate optimization of the model with less training data.

## Supplemental Information

10.7717/peerj-cs.916/supp-1 Supplemental Information 1 Video content captioning model structure. The model includes three parts: feature extraction, video captioning generation, reward mechanism (Policy Gradient). *L* is the number of transformer encoder blocks in the encoder of the model, *w^s^* is the word sequence generated by the model, and r(*) is the reinforcement learning reward function

10.7717/peerj-cs.916/supp-2 Supplemental Information 2 Captioning content generation module structure composed of Transformer and LSTM.

10.7717/peerj-cs.916/supp-3 Supplemental Information 3 Encoder block structure in VIT. Specifically, the encoding block first inputs the features into Layer Norm and Multi-Head Attention, and then sends them to the MLP block.

10.7717/peerj-cs.916/supp-4 Supplemental Information 4 LSTM network model.

10.7717/peerj-cs.916/supp-5 Supplemental Information 5 Reinforcement Learning network model.

10.7717/peerj-cs.916/supp-6 Supplemental Information 6 Visual comparison of video content captioning examples generated by our model. GT means real annotated captioning.

## Additional Information and Declarations

### Data Availability

The following information was supplied regarding data availability: The dataset MSR-VTT was published in Xu et al. (2016): https://www.microsoft.com/en-us/research/publication/msr-vtt-a-large-video-description-dataset-for-bridging-video-and-language/ and is available at GitHub: https://github.com/caidhome/video-captioning/tree/master.
