# Supplementary figures and images for: Video captioning based on vision transformer and reinforcement learning
Source: PeerJ Comput Sci. 2022 Mar 16;8:e916. doi: 10.7717/peerj-cs.916 (PMC9044334; doi:10.7717/peerj-cs.916)

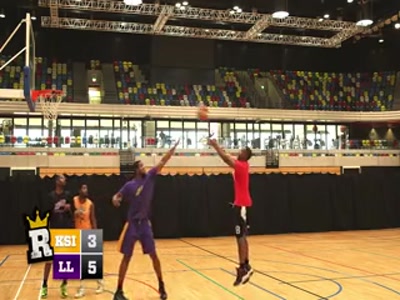

Supplement: Supplemental Information 1 — The model includes three parts: feature extraction, video captioning generation, reward mechanism (Policy Gradient). L is the number of transformer encoder blocks in the encoder of the model, ws is the word sequence generated by the model, and r(*) is the reinforcement learning reward function [file peerj-cs-08-916-s001.vsdx › visio/media/image2.jpeg]

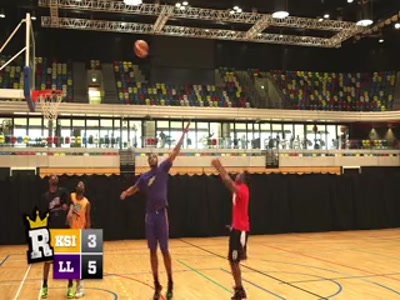

Supplement: Supplemental Information 1 — The model includes three parts: feature extraction, video captioning generation, reward mechanism (Policy Gradient). L is the number of transformer encoder blocks in the encoder of the model, ws is the word sequence generated by the model, and r(*) is the reinforcement learning reward function [file peerj-cs-08-916-s001.vsdx › visio/media/image4.jpeg]

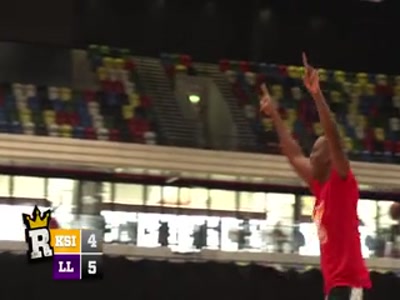

Supplement: Supplemental Information 1 — The model includes three parts: feature extraction, video captioning generation, reward mechanism (Policy Gradient). L is the number of transformer encoder blocks in the encoder of the model, ws is the word sequence generated by the model, and r(*) is the reinforcement learning reward function [file peerj-cs-08-916-s001.vsdx › visio/media/image9.jpeg]

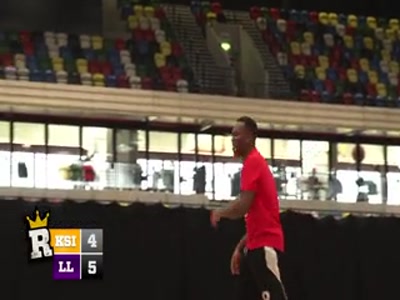

Supplement: Supplemental Information 1 — The model includes three parts: feature extraction, video captioning generation, reward mechanism (Policy Gradient). L is the number of transformer encoder blocks in the encoder of the model, ws is the word sequence generated by the model, and r(*) is the reinforcement learning reward function [file peerj-cs-08-916-s001.vsdx › visio/media/image10.jpeg]

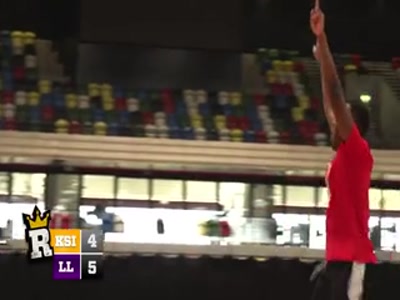

Supplement: Supplemental Information 1 — The model includes three parts: feature extraction, video captioning generation, reward mechanism (Policy Gradient). L is the number of transformer encoder blocks in the encoder of the model, ws is the word sequence generated by the model, and r(*) is the reinforcement learning reward function [file peerj-cs-08-916-s001.vsdx › visio/media/image8.jpeg]

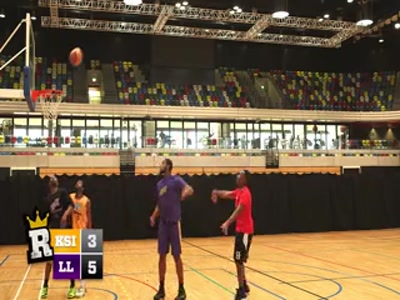

Supplement: Supplemental Information 1 — The model includes three parts: feature extraction, video captioning generation, reward mechanism (Policy Gradient). L is the number of transformer encoder blocks in the encoder of the model, ws is the word sequence generated by the model, and r(*) is the reinforcement learning reward function [file peerj-cs-08-916-s001.vsdx › visio/media/image6.jpeg]

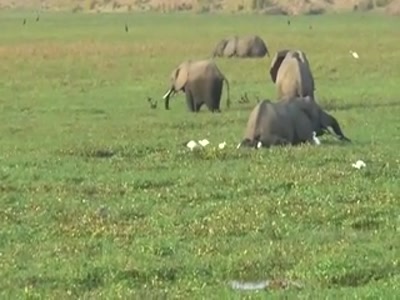

Supplement: Supplemental Information 6 [file peerj-cs-08-916-s006.vsdx › visio/media/image1.jpeg]

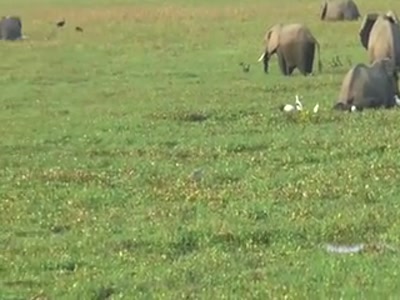

Supplement: Supplemental Information 6 [file peerj-cs-08-916-s006.vsdx › visio/media/image2.jpeg]

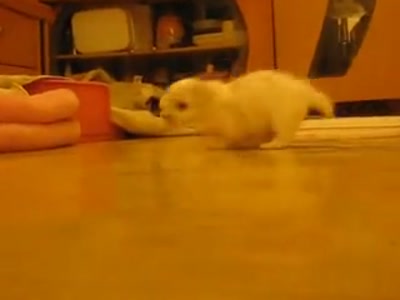

Supplement: Supplemental Information 6 [file peerj-cs-08-916-s006.vsdx › visio/media/image3.jpeg]

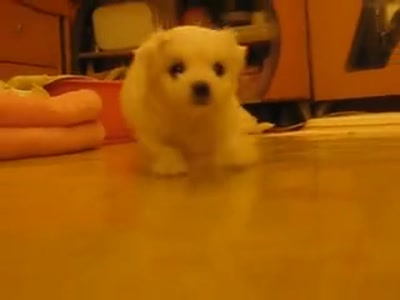

Supplement: Supplemental Information 6 [file peerj-cs-08-916-s006.vsdx › visio/media/image4.jpeg]

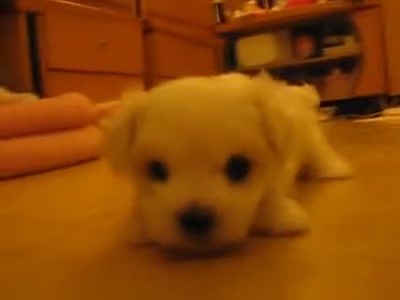

Supplement: Supplemental Information 6 [file peerj-cs-08-916-s006.vsdx › visio/media/image5.jpeg]

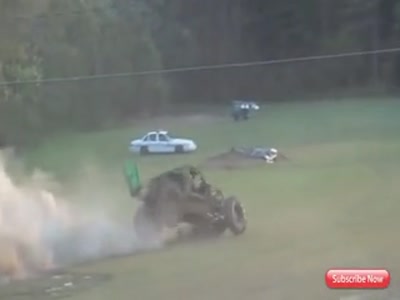

Supplement: Supplemental Information 6 [file peerj-cs-08-916-s006.vsdx › visio/media/image6.jpeg]

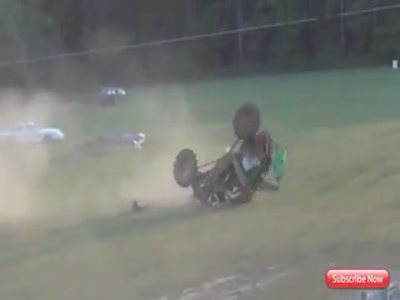

Supplement: Supplemental Information 6 [file peerj-cs-08-916-s006.vsdx › visio/media/image7.jpeg]

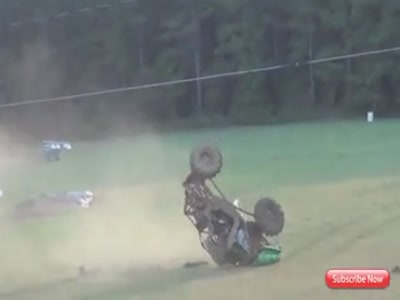

Supplement: Supplemental Information 6 [file peerj-cs-08-916-s006.vsdx › visio/media/image8.jpeg]

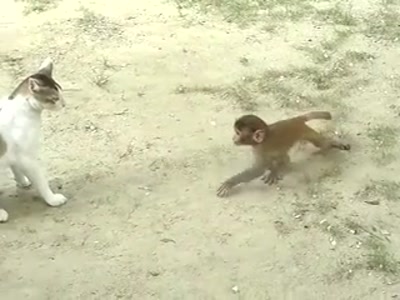

Supplement: Supplemental Information 6 [file peerj-cs-08-916-s006.vsdx › visio/media/image9.jpeg]

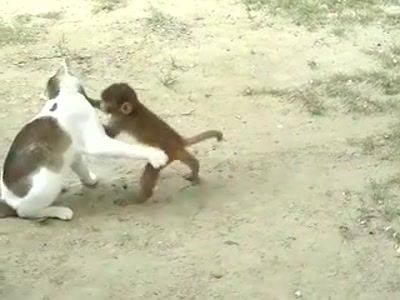

Supplement: Supplemental Information 6 [file peerj-cs-08-916-s006.vsdx › visio/media/image10.jpeg]

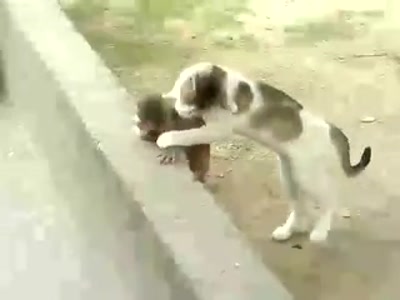

Supplement: Supplemental Information 6 [file peerj-cs-08-916-s006.vsdx › visio/media/image11.jpeg]

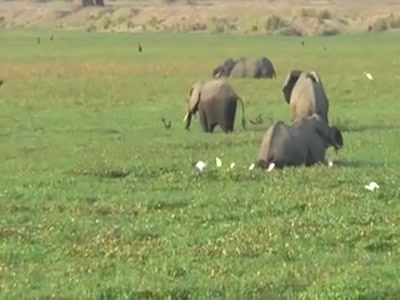

Supplement: Supplemental Information 6 [file peerj-cs-08-916-s006.vsdx › visio/media/image12.jpeg]
